# Proteomic analysis of the response of the plant growth-promoting bacterium *Pseudomonas putida *UW4 to nickel stress

**DOI:** 10.1186/1477-5956-7-18

**Published:** 2009-05-07

**Authors:** Zhenyu Cheng, Yi-Yun C Wei, Wilson WL Sung, Bernard R Glick, Brendan J McConkey

**Affiliations:** 1 Department of Biology, University of Waterloo, 200 University Avenue West, Waterloo, Ontario N2L 3G1, Canada

## Abstract

**Background:**

Plant growth-promoting bacteria can alleviate the inhibitory effects of various heavy metals on plant growth, via decreasing levels of stress-induced ethylene. However, little has been done to detect any mechanisms specific for heavy metal resistance of this kind of bacteria. Here, we investigate the response of the wild-type plant growth-promoting bacterium *Pseudomonas putida *UW4 to nickel stress using proteomic approaches. The mutant strain *P. putida *UW4/AcdS^-^, lacking a functional 1-aminocyclopropane-1-carboxylic acid deaminase gene, was also assessed for its response to nickel stress.

**Results:**

Two dimensional difference in-gel electrophoresis (DIGE) was used to detect significantly up- or down- regulated proteins (*p *< 0.05, | ratio | > 1.5) in *P. putida *in response to the presence of 2 mM Ni. Out of a total number of 1,702 proteins detected on the analytical gels for *P. putida *UW4, the expression levels of 82 (4.82%) proteins increased significantly while the expression of 81 (4.76%) proteins decreased significantly. Of 1,575 proteins detected on the analytical gels for *P. putida *UW4/AcdS^-^, the expression levels of 74 (4.70%) proteins increased and 51 (3.24%) proteins decreased significantly. Thirty-five proteins whose expression was altered were successfully identified by mass spectrometry and sequence comparisons with related species. Nineteen of the identified proteins were detected as differentially expressed in both wild-type and mutant expression profiles.

**Conclusion:**

Functional assessment of proteins with significantly altered expression levels revealed several mechanisms thought to be involved in bacterial heavy metal detoxification, including general stress adaptation, anti-oxidative stress and heavy metal efflux proteins. This information may contribute to the development of plant growth-promoting bacteria mediated phytoremediation processes.

## Background

Small amounts of nickel are essential for the functioning of a number of nickel-containing enzymes including hydrogenase, urease, carbon monoxide dehydrogenase, and superoxide dismutase [1]. However, nickel is one of the most common metal contaminants in the environment and is often toxic to bacteria at high concentrations. This toxicity is generally a consequence of nickel binding to sulfhydryl groups of sensitive enzymes or displacing essential metal ions in a variety biological processes [2]. Also, cationic nickel (mostly Ni^2+^) can cause a significant oxidative stress in bacteria by facilitating of the production of oxidized bis-glutathione, which releases hydrogen peroxide [2]. In bacterial cells, cation efflux-mediated nickel resistance is one of the best-known mechanisms of nickel detoxification [3,4]. In addition, the up-regulation of genes encoding anti-oxidant enzymes is often the main response of many bacteria to various metals [5]. In this regard, thiol-containing molecules were shown to be capable of detoxifying cadmium in *Rhizobium leguminosarum bv. viciae *[6] and nickel in human cells [7].

Recently, researchers have attempted to develop metal phytoremediation protocols including the harvesting and combusting of plants grown in metal-contaminated soil, as an alternative to the traditional remediation methods that involve excavation and removal of soil to secured landfill sites. Ideally, the plants used for metal phytoremediation grow rapidly and produce high levels of biomass. Unfortunately, plant growth, even plants that are relatively metal tolerant, is generally inhibited in the presence of high concentrations of metals. One of the strategies that have been used to overcome this problem is the addition of ACC deaminase-containing plant growth-promoting bacteria (PGPB) that can improve plant performance under various environmentally stressful conditions. The pyridoxal phosphate enzyme ACC (1-aminocyclopropane-1-carboxylic acid) deaminase [EC 4.1.99.4] catalyzes the deamination of ACC to produce α-ketobutyrate and ammonia. ACC is the immediate precursor of the phytohormone ethylene, which becomes elevated as a consequence of various environmental stresses [8,9] and is an important mediator of plant stress responses. ACC deaminase-containing PGPB attached to plant host surfaces can act as a sink for ACC, thereby allowing plants to maintain a beneficial level of ethylene without the risk of reaching inhibitory levels [8,9]. By limiting the deleterious ethylene level that might otherwise be generated in plants in response to the presence of high levels of metals, PGPB containing ACC deaminase can dramatically increase plant biomass in the presence of a variety of heavy metals both in the laboratory [10-16] and in the field [17,18]. However, the mutant strain *P. putida *UW4/AcdS^-^, which lacks the *acdS *gene encoding ACC deaminase and is in turn responsible for modulating stress ethylene levels, does not promote plant growth to the same extent [19]. Although it is expected that the reduced impact of the *P. putida *UW4/AcdS^- ^strain on plant growth is mostly likely due to higher levels of ethylene, the mutant strain was also compared to verify that the *P. putida *UW4 response to nickel stress was largely independent of the *acdS *gene.

Proteomic techniques such as 2-D (2 dimensional) gel electrophoresis and mass spectrometry may be used to characterize and quantify bacterial responses to environmental stimuli. In particular, DIGE is a method that can be used to accurately quantify protein expression differences under various conditions [20]. Here, differences in expression levels in the proteome of the wild-type PGPB *P. putida *UW4 and mutant *P. putida *UW4/AcdS^- ^were examined. This work further facilitates an understanding of the biochemical basis of bacterial resistance to nickel stress, which for *P. putida *is an important component of its ability to facilitate phytoremediation of heavy metals in soil.

## Results

### Bacterial growth

The growth rates of wild-type *P. putida *UW4 and mutant *P. putida *UW4/AcdS^- ^in rich medium (TSB) without nickel at their optimal growth temperature (30°C) were 0.55 and 0.63 generation/hour, respectively (Table 1). These rates were reduced to 44% and 40% of the control level when growth was in TSB containing 2 mM nickel, and dropped to 15% and 14% in the presence of 5 mM nickel. The inhibitory effects of different concentrations of nickel on the growth rates of the wild type and mutant strains did not notably differ from each other (p > 0.05).

**Table 1:** The effects of nickel on the growth rates of *P. putida *UW4 wild-type and mutant strains.

Bacterial strains	*P. putida *UW4			*P. putida *UW4/*acdS*^-^		
Added nickel	0 mM	2 mM	5 mM	0 mM	2 mM	5 mM
Growth rate (h^-1^)	0.55 ± 0.02	0.24 ± 0.03	0.08 ± 0.01	0.63 ± 0.05	0.25 ± 0.04	0.09 ± 0.01
Growth rate (% of control)	100	44*	15*	100	40*	14*

* Denotes values determined not to be significantly different between the wild type and the mutant growth rate values by independent t-test (p > 0.05). Growth rates were measured in duplicate.

### Protein expression profiles

The expression profiles of wild-type *P. putida *UW4 and the mutant UW4/AcdS^- ^in response to 2 mM of nickel were analyzed. Of a total of 1,702 proteins detected on the analytical gels for wild-type *P. putida *UW4, the expression levels of 82 (4.82%) proteins increased significantly and 81 (4.76%) proteins decreased significantly (p < 0.05, | Ratio | > 1.5). Results were similar for the *P. putida *UW4/AcdS^- ^strain, with 1,575 proteins detected on the analytical gels. In this case, the expression levels of 74 (4.70%) proteins increased significantly and 51 (3.24%) proteins decreased significantly.

**Figure 1:**
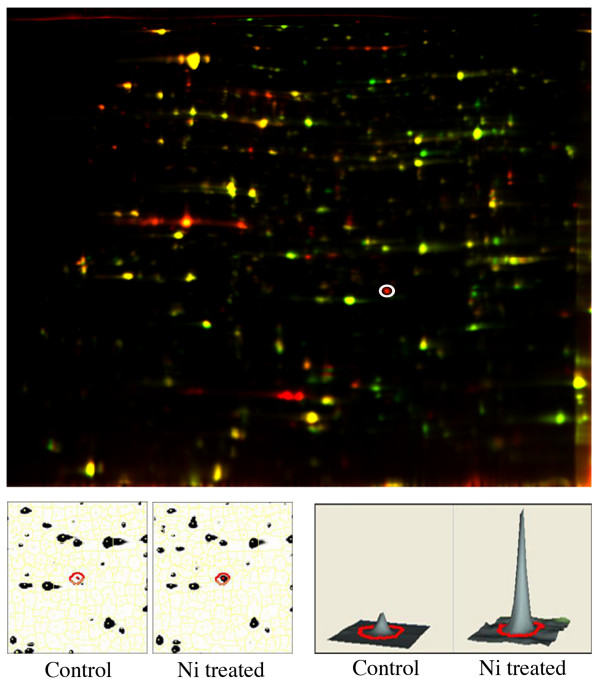
**(top) An analytical gel comparing protein expression of the bacterium *P. putida *UW4 exposed to 2 mM of nickel (red) and control (green)**. (bottom) The "spot view" and the "volume view" from a standard DeCyder analysis showing a sample up-regulated protein, general stress protein CTC.

Figure 1 (top) shows a representative analytical gel comparing protein expression of wild-type *P. putida *UW4 in the presence and absence of 2 mM nickel. On this gel, the 2 mM nickel treated sample is labeled with Cy5 (red) and the control labeled with Cy3 (Green), and up- regulated and down- regulated proteins appear as red and green spots respectively. A sample up-regulated protein, general stress protein CTC, is circled in Figure 1. The "spot view" (lower left) shows the enlarged gel area surrounding the highlighted spot for Cy3 and Cy5 channels, with corresponding volume calculations for each channel (lower right). The expression of the highlighted protein was increased 3.56-fold in the presence of nickel compared to the control, as calculated based on peak volume.

### Mass spectrometric analysis

**Figure 2:**
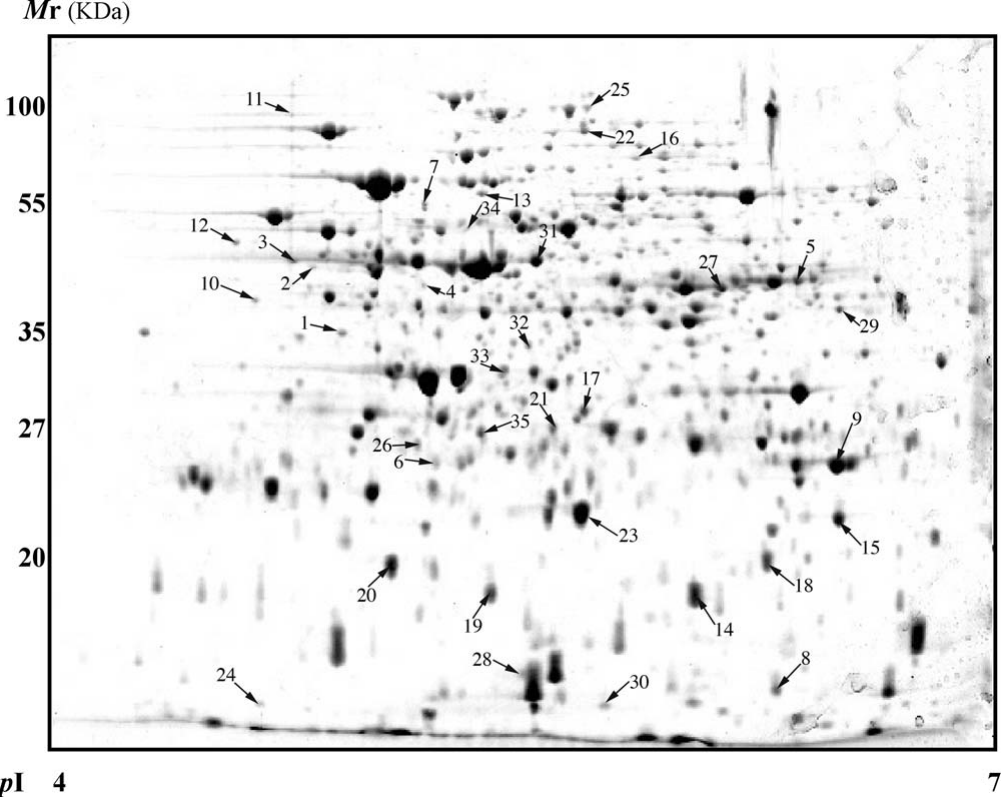
**A preparative gel of *P. putida *UW4 control proteins**. About 1 mg of protein samples were loaded onto IPG 4–7 strip in the first dimension, and separated using 12% SDS-PAGE gel in the second dimension. Proteins spots were visualized by Coomassie staining. The 35 identified and labeled spots are annotated according to the numbering in Table 2.

All protein spots identified as having significant changes expression levels and present in sufficient amounts to be visible on a coomassie-stained preparative gel were excised for mass spectrometric analysis. From the excised protein spots, a total of 35 proteins with significantly differential expression levels were identified by mass spectrometry (Table 2, Figure 2). Although the genomic sequence of *P. putida *UW4 has not yet been characterized, proteins were able to be identified via homologous proteins primarily from other *Pseudomonas *strains by the PEAKS software, which combines *de novo *peptide sequencing with database identifications (Table 2). Among these 35 proteins, the best sequence coverage (50.5%) was obtained from the protein identified as ion/magnesium superoxide dismutase by database match with an homologous *P. fluorescens *protein. The protein ArsA and the immunodominant antigen B were identified via matches to proteins from *Bradyrhizobium *and *Staphylococcus*, respectively.

**Table 2:** Differentially expressed proteins identified in *P. putida *UW4 wild-type (WT) and mutant strains, grouped by putative functional role.

Protein*	**Accession number^†^**	**MW(kDa)/p*I*^§^**	**Score/Sequence coverage %^¶^**	**WT Ratio^‡^**	**Mut Ratio^‡^**
**Amino acid metabolism**					
1. ACC deaminase	Q4KK38_PSEF5	35.1/5.37	84.15/9.06	1.58	N/A
2. diaminopimelate decarboxylase	Q3K4R9_PSEPF	45.1/4.97	93.68/10.6	10.3	2.80
3. porphyromonas-type peptidyl-arginine deiminase	Q3KJM1_PSEPF	40.7/4.81	40.07/8.7	2.08	1.54
4. arginine biosynthesis protein	Q3K7U0_PSEPF	42.6/5.16	96.15/8.89	-3.05	-2.48
5. aminotransferase	Q3KG66_PSEPF	43.3/6.01	98.92/19.14	-2.32	-2.87
6. 3-isopropylmalate dehydratase	Q3KF23_PSEPF	24.2/5.39	87.37/35.98	N/S	-1.79
7. anthranilate synthase	Q4K4Z5_PSEF5	54.2/5.08	92.94/15.96	N/S	-3.25
**General stress**					
8. universal stress protein	Q4KFC6_PSEF5	16.2/5.95	78.23/20.69	1.99	1.80
9. general stress protein CTC	Q3K6W3_PSEPF	21.2/5.99	98.62/16	3.56	2.08
**Transport/efflux**					
10. major outer membrane protein	Q9X4L6_PSEFL	36.5/4.68	90.82/13.08	2.00	2.15
11. outer membrane protein OMP85	Q4KHG8_PSEF5	87.5/5.21	128/20^¶^	3.58	1.56
12. porin D	Q48FB5_PSE14	46.9/4.94	60.66/4.32	2.44	3.66
13. ArsA	Q89K44_BRAJA	63.5/5.81	99/4	1.89	1.68
**Protein synthesis and folding**					
14. peptidyl-prolyl cis-trans isomerase B	Q87YQ0_PSESM	18.2/5.86	71.15/6.59	-1.62	1.55
15. ribosome recycling protein	Q886P0_PSESM	20.4/6.76	46.7/8.65	-2.16	-1.51
16. glutaminyl-tRNA synthetase	Q1IBQ0_9PSED	64.5/5.62	91.43/10.41	-2.29	N/S
**Cell cycle and communication**					
17. MinD	Q3KFL9_PSEPF	33.7/5.55	99.17/29.7	-1.59	-1.51
18. DNA-binding protein	Q889U1_PSESM	20.9/5.88	46.84/16.93	-2.27	N/S
19. transcription regulator TraR/DksA family	Q3K6R3_PSEPF	16.8/5.21	62.4/20.41	-1.59	N/S
**Anti-oxidative**					
20. thiol specific antioxidant	Q3KD94_PSEPF	17.6/4.99	87.21/30.72	3.38	N/S
21. ferredoxin NADP-reductase	Q48FA2_PSE14	29.6/5.32	97.54/29.73	1.69	1.77
22. GTP-binding protein TypA	Q3KJG5_PSEPF	67.0/5.40	95.1/9.9	2.32	1.5
23. superoxide dismutase	Q3K7N1_PSEPF	22.0/5.56	99.86/50.51	5.62	4.63
24. thioredoxin	Q3K4W0_PSEPF	11.7/5.06	95.81/44.04	19.8	3.41
25. 2-oxo-acid dehydrogenase	Q3KJ49_PSEPF	99.4/5.48	98.77/9.31	N/S	1.73
**Other**					
26. ubiquinone dehydrogenase	Q3KA62_PSEPF	25.3/5.16	73.16/22.77	N/S	-1.93
27. thiolase	Q4ZY90_PSEU2	40.4/5.91	89.57/6.89	-1.82	N/S
28. similar to immunodominant antigen B	Q4L351_STAHJ	18.3/5.08	48.52/10.56	29.6	N/S
29. aldo/keto reductase	Q3K722_PSEPF	38.9/5.88	69.66/12.72	-1.61	N/S
30. pterin-4-alpha-carbinolamine dehydratase	Q3KG65_PSEPF	13.3/5.83	99.4/78.81	-2.91	2.08
31. isocitrate dehydrogenase	Q4K9U5_PSEF5	45.3/5.27	99.97/27.75	-1.77	N/S
32. transaldolase	Q3K9H0_PSEPF	33.7/5.66	96.25/15.26	-2.8	N/S
33. dihydrodipicolinate synthase	Q3KGJ2_PSEPF	31.3/5.77	97.86/39.04	-1.83	N/S
34. hypothetical protein	Q3KFH8_PSEPF	55.6/5.51	82.14/5.87	2.01	1.52
35. hypothetical protein	Q3KI45_PSEPF	25.7/5.36	83.22/12.61	N/S	-1.51

* Protein numbering refer to numbers in Figure 2.

† Accession number of top database match from the *UniprotKB *database.

§MW and p*I *were calculated from amino acid sequence and compared with gel location.

¶Score and sequence coverage were calculated by PEAKS software except for the underlined value, which used MASCOT scores. All identifications were confirmed using MASCOT MS/MS ion search and/or peptide-fingerprinting and significant matches (*p *< 0.05) were retained.

‡ Ratios were calculated as R = (treated/control) or R = -(control/treated) for up-regulated and down-regulated proteins respectively. N/S, expression was not significantly altered; N/A, not applicable. All ratios shown are statistically significant (*p *< 0.05).

The functional annotation of these proteins revealed a variety of cellular functions and can be divided into 7 categories (Table 2). Out of the 35 identified proteins, nineteen were common to both wild-type and the mutant expression profiles. Eleven proteins were identified as having significantly different expression only in the wild-type profile and five proteins had altered expression only in the mutant profile. The "WT ratio" and "Mut ratio" represent the protein expression changes in response to 2 mM nickel.

ACC deaminase was identified in the wild-type *P. putida *UW4 profile where its expression was increased by 1.58 fold in response to 2 mM nickel (Table 2). Not surprisingly, there was no data available for its expression in the mutant strain *P. putida *UW4/AcdS^- ^profile since the gene encoding ACC deaminase was deleted in this strain. The largest expression change was in the wild-type *P. putida *UW4 profile in the protein matching immunodominant antigen B where the increase was nearly 30-fold in response to 2 mM nickel (Table 2). This protein was not detected as significant in the mutant expression profile.

Surprisingly, the expression of peptidyl-prolyl cis-trans isomerase B and pterin-4-alpha-carbinolamine dehydratase were altered in opposite directions between the wild-type and mutant profiles, and both had relatively small changes in expression. While it is possible these may represent real differences between the two tested strains, they could simply represent artifacts or false positives within the set false discovery rate of 5%.

The nineteen proteins that were found in both the wild-type and the mutant expression profiles, and were changed in a similar manner in both strains, appear to be related to nickel stress and detoxification. Proteins involved in amino acid synthesis, such as arginine biosynthesis protein and aminotransferase were both down regulated, as was a ribosome-recycling protein, which is also involved in protein synthesis. In addition, MinD, a protein essential for cell division was also down regulated. On the other hand, transport proteins (both import and export), such as major outer membrane protein, outer membrane protein OMP85, porin D and ArsA, were all up regulated. The other up-regulated proteins were categorized as either general stress proteins, such as universal stress protein and general stress protein CTC, or anti-oxidative proteins such as ferredoxin NADP-reductase, GTP-binding protein TypA, superoxide dismutase, and thioredoxin. Among these stress-related proteins, superoxide dismutase expression was increased by 5.62-fold and 4.63-fold in the wild-type and the mutant respectively, and thioredoxin was increased by 19.84-fold in the wild-type and by 3.41-fold in the mutant.

## Discussion

In spite of the lack of genomic information regarding *P. putida *UW4, proteins were identified by mass spectrometric analyses with high confidence. The quantitative proteomic analysis of differential protein expression profiles utilized in this work was effective in identifying both comprehensive and biologically significant bacterial responses to environmental factors.

More specifically, the effects of nickel on growth and protein expression were very similar for wild-type *P. putida *UW4 and mutant *P. putida *UW4/AcdS^-^. Although the growth rate of the mutant strain was slightly faster than the growth rate of the wild-type in the absence of nickel, they decreased to a similar extent in the presence of two different concentrations of nickel. More than half of the identified protein expression changes in the presence of nickel occurred in both wild-type and mutant strains, consistent with the *P. putida *UW4 ACC deaminase enzyme not having a direct role in the resistance of the bacterium to nickel toxicity. Similar results have been found previously, in which alterations to the non-essential endogenous ACC deaminase gene did not noticeably change the physiology of *P. putida *UW4 [19,21]. As expected, ACC deaminase was only present in the wild-type expression profile; it was slightly up regulated in the presence of 2 mM nickel. No other notable changes were observed, suggesting that interactions between nickel response and ACC deaminase activity are minimal.

Interestingly, the expression of the protein spot matched with Q4L351_STAHJ was increased almost 30-fold in the wild-type *P. putida *UW4 in response to 2 mM nickel (Table 2). The identified match for this protein was a *Staphylococcus haemolyticus *protein annotated as 'similar to immunodominant antigen B' with a relatively low score (48.52%). *S. haemolyticus *is not closely related to *P. putida *UW4, and *S. haemolyticus *is Gram-positive whereas *P. putida *UW4 is Gram-negative. However, three peptides were matched with approximately 10% sequence coverage to Q4L351_STAHJ. And the observed molecular weight (18.3 kDa) and p*I *(5.08) of this protein on the gel matched well with the calculated values of Q4L351_STAHJ protein (18.4 kDa, p*I *5.01). In addition, the possibility of *S. haemolyticus *contamination in the original culture is unlikely since 33 out of 35 proteins were identified as *Pseudomonas *proteins. The *P. putida *UW4/AcdS^- ^mutant was constructed by specifically disrupting the ACC deaminase gene using homologous recombination [19], so that is possible that regulation of the this protein may be affected by ACC deaminase, a protein whose expression is regulated by a variety of factors in a complex manner [22-24]. Very little is known about the function of this *S. haemolyticus *protein. An NCBI Blast analysis of this protein found no known homologous proteins in *Pseudomonas *species, and relatively few related proteins in other *Staphylococcus *species. Previous work has shown that another immunodominant antigen identified in *Burkholderia cepacia *functioned as efflux pump [25]. It is likely that the identified protein in *P. putida *UW4 is also a cell surface protein, and may be involved in import/export functions, but this is conjecture at this point. It is possible that the gene coding for this protein was acquired by *P. putida *UW4 by lateral gene transfer, a common mechanism for the acquisition of *acdS *and other genes by *pseudomonads *[26]. The ArsA protein that was identified via a match to a *Bradyrhizobium *protein may also have been obtained by *P. putida *UW4 via a similar mechanism.

As expected, bacterial cells responded to nickel stress by decreasing expression of proteins involved in cellular activities, such as amino acid synthesis, protein synthesis and folding, DNA replication, cell division and cell communication. Proteins involved in these processes were all down regulated when cells were exposed to nickel stress. Proteins involved in general, non-specific importation of metabolites into the cell were up-regulated, although this could possibly result in the intensification of the toxic effects of the nickel.

In addition, bacterial universal stress protein and general stress protein CTC, which were previously reported to be induced by and responsible for the resistance to various stresses [27-29], were also up regulated in both strains and presumably participate in the nickel resistance response. An efflux protein ArsA, which is involved in arsenate exportation [3], was also up regulated in both strains. Although it was only one protein of many that are responsible for efflux-mediated detoxification of arsenate, the up regulation of this protein suggests that a similar efflux-mediated mechanism may be involved in nickel detoxification in *P. putida *UW4.

In Gram-negative bacteria, heavy metal cations can bind to glutathione and the resulting products (bisglutathione complex) tend to react with molecular oxygen to form oxidized bisglutathione, releasing the metal cation and hydrogen peroxide [2]. Since bisglutathione must be reduced in an NADPH-dependent reaction and the released metal cations immediately begin another cycle of binding and oxidation, this can cause considerable oxidative stress. Here, a variety of anti-oxidative proteins were up regulated in both strains in the presence of 2 mM nickel. In particular the expression of thioredoxin was increased almost 20-fold (Table 2). All anti-oxidative proteins that were observed to be up regulated in *P. putida *UW4 were previously shown to be responsible for anti-oxidative stress and/or up regulated in the presence of the oxidative stress [30-37]. Both the numbers of the proteins that were up regulated and the magnitude of their changes suggested that the production of anti-oxidative stress proteins was a major response of *P. putida *UW4 to the presence of nickel. Other studies have also suggested that these proteins are involved in nickel detoxification [30-37].

In this study, two hypothetical proteins with altered expression levels were identified and one of them was up regulated in both the wild-type and mutant. The NCBI Blast search of the hypothetical protein with accession number Q3KFH8_PSEPF matches a number of other hypothetical proteins, but none has been functionally annotated. The other hypothetical protein, with accession number Q3KI45_PSEPF, matches a putative *Pseudomonas *signal peptide with a 72% identity and a predicted *Pseudomonas *periplasmic/secreted protein with a 60% identity. In both cases, the hypothetical proteins may be involved in environmental signal transduction. In any event, additional studies focused on these genes may facilitate a better understanding of the mechanisms involved in bacterial heavy metal resistance.

## Conclusion

Plant growth promoting bacteria containing ACC deaminase have been previously shown to alleviate the inhibitory effects of various heavy metals on plant growth [8-16]. However, how the bacteria react to the heavy metals stresses has not been previously characterized. In this work, an examination of the proteome of both the wild-type *P. putida *UW4 and the mutant *P. putida *UW4/AcdS^- ^revealed systematic nickel resistance responses of this bacterium including general stress adaptation, anti-oxidative stress and heavy metal efflux, which may be useful in the development of PGPB-mediated phytoremediation protocols.

## Methods

### Bacterial strains and growth

*Pseudomonas putida *UW4 was originally isolated from the rhizosphere of common reeds and was initially classified as *Pseudomonas *sp. [38]. It was later reclassified as *Enterobacter cloacae *based on fatty acid analysis [39], and more recently as *Pseudomonas putida *based on 16S rDNA sequence analyses and metabolic activity [26]. The mutant strain *P. putida *UW4/AcdS^- ^was constructed by disrupting the ACC deaminase gene (*acdS*). A tetracycline resistance gene was inserted within the coding region of the *acdS *gene by homologous recombination [19]. The wild-type and mutant strains were cultivated aerobically in Tryptic Soy broth (TSB; Fisher Scientific Co.) or plates at 30°C. An estimate of the tolerance of these bacterial strains to nickel was assessed by culturing them in the presence of 0, 2 or 5 mM nickel.

### Bacterial treatment and protein extraction

*P. putida *UW4 and the mutant were grown to late-log phase in 50 mL of TSB medium or TSB medium supplemented with 2 mM nickel. Bacterial cells were harvested by centrifugation at 8,000 *g *at 4°C for 10 minutes. The pellets were washed twice with cold water, weighed and then stored at -70°C overnight. The cell pellet was thawed on ice for 15 minutes and resuspended in ice-cold lysis buffer (2 M thiourea, 7 M urea, 4% CHAPS, 30 mM Tris pH 8.5; 5 mL/g wet cells) containing 100 μL of protease inhibitor cocktail (Sigma-Aldrich Canada Ltd., Oakville, Ontario). The cells were then stirred at 4°C for 30 minutes. The lysate was centrifuged at 10,000 *g *at 4°C for 10 minutes, followed by centrifugation at 150,000 *g *at 4°C for 90 minutes. The final supernatant was saved as the whole cell extract and stored at -70°C until use.

### Proteomic profiling and difference in-gel electrophoresis (DIGE)

The DIGE analyses were performed according to the supplier's instructions (GE Healthcare, Mississauga, ON). Fifty μg of each sample was labeled with 200 pmol of different CyDye DIGE fluors. After labeling, all three samples (internal standard, control sample, treated sample) were mixed together and loaded on Immobiline DryStrips (pH 4–7, 24 cm). Isoelectric focusing was performed using an Ettan IPGphor II (GE Healthcare, Mississauga, ON). The second dimension was run on 12% SDS-PAGE gels and the analytical gels were scanned on a Typhoon 9400 scanner (GE Healthcare, Mississauga, ON). Each analysis was done in triplicate. Differential expression profiles were analyzed using DeCyder V 6.0 software (GE Healthcare, Mississauga, ON). Ratios are based on standardized volumes of protein spots between the Ni-treated and control bacteria. Expression ratios were calculated by the DeCyder software as R = (treated/control) for up-regulated proteins, and calculated as R = -(control/treated) for down-regulated proteins, where a 2-fold up-regulation or down-regulation is represented by 2 and -2, respectively. Statistical significance was calculated using the DeCyder software and corrected for multiple hypothesis testing using the FDR option.

Preparative 2-D gels of samples intended for mass spectrometry, and loaded with 1.0 mg protein were stained overnight with Bio-Safe Coomassie (Bio-Rad Laboratories, Hercules, CA) and destained with water. Spots of interest were excised from the gel, the gel pieces were washed with water and destained with 50 mM NH_4_HCO_3_/50% acetonitrile (ACN). Proteins were reduced by incubation with 10 mM dithiothreitol in 100 mM NH_4_HCO_3 _at 50°C for 30 min, and then alkylated by incubation with 55 mM iodoacetamide in 100 mM NH_4_HCO_3 _for 30 min in the dark. After being dehydrated with 100% ACN and air-dried, the gel pieces were rehydrated for ten minutes in a trypsin solution (Promega Corporation, Madison, Wisconsin) in a ratio of approximately 1:10 (w/w) of trypsin:protein. Fifty μL of 50 mM NH_4_HCO_3 _(pH 8.0) was added to each gel piece and the proteins were digested at 37°C for 18 hours. The peptides were extracted by vortexing and then concentrated to 10 μL in a Savant SpeedVac. The samples were cleaned using a C-18 ZipTip system (Millipore) and eluted with 5 μL of 50% ACN. One μL of 1% formic acid was added to the eluate to protonate the peptides. Mass spectrometry was performed using a Waters Micromass quadrupole time of flight (Q-TOF) Ultima using nano-spray injection as the sample delivery method. All proteins except one were identified by the PEAKS software 3.1 [40] (Bioinformatics Solutions Inc., Waterloo, ON), which combines auto *de novo *sequencing and database searching. Within the PEAKS software the parental and fragment mass error were 0.2 Da and 0.1 Da, respectively, and trypsin was set as the digestion enzyme with one missed cleavage allowed. Carbamidomethylation and methionine oxidation were set as the fixed and variable post-translational modifications, respectively. The MASCOT peptide-fingerprinting algorithm [38] was used in parallel with PEAKS, with the same parameters used for the digestion enzyme, post-translational modifications, and missed cleavages as with the PEAKS software. Default peptide tolerances were used. The non-redundant protein sequence database MSDB (Imperial College, London) was used as the target database for PEAKS and MASCOT. MSDB combines sequences from PIR, TrEMBL, GenBank, and SwissProt. MSDB was designed specifically for mass spectrometry applications and is distributed with the MASCOT search engine [41]. Identifications were confirmed using the MASCOT MS/MS ion search and/or peptide-fingerprinting algorithm [41], and only significant hits (*p *< 0.05) were retained in the list of identifications.

## Competing interests

The authors declare that they have no competing interests.

## Authors' contributions

ZC carried out the experiments and drafted the manuscript. ZC, YYCW, and WWLS conducted sample preparation and analysis. BRG and BJM participated in the design of the study and drafted the manuscript. All authors have read and approved the final manuscript.
